# Perception and practice of breastfeeding among HIV positive mothers receiving care for prevention of mother to child transmission in South-East, Nigeria

**DOI:** 10.1186/s13006-018-0191-8

**Published:** 2018-11-28

**Authors:** Ancilla-Kate Umeobieri, Chinyere Mbachu, Benjamin S. C. Uzochukwu, Aniwada Elias, Babatunde Omotowo, Chuka Agunwa, Ikechukwu Obi

**Affiliations:** 1 Department of Community Medicine, College of Medicine, University of Nigeria Enugu campus, Enugu, Nigeria; 2 Institute of Public Health, University of Nigeria Enugu campus, Enugu, Nigeria

**Keywords:** HIV, Prevention of mother-to-child transmission, Breastfeeding, Perception, Practice

## Abstract

**Background:**

Although the risk of HIV transmission through breastfeeding is reduced considerably with the use of antiretroviral therapy, infant feeding by HIV positive mothers remains controversial. Weighing risks against benefits generates intense debate among policymakers, program managers and service providers in sub-Saharan Africa, considering that the major causes of infant death of malnutrition and infectious diseases, could be prevented if mothers breastfeed their babies. Whereas breastfeeding involves some risk of HIV transmission, not breastfeeding poses considerable risk to infant survival. This study investigated perceptions and practice of breastfeeding of HIV-exposed infants among HIV positive mothers.

**Methods:**

A cross-sectional descriptive study was conducted in Enugu metropolis among HIV positive mothers receiving care for prevention of mother-to-child transmission of HIV from two public and two private hospitals. Interviewer-administered questionnaire survey was done with 550 participants as they exited the final point of service delivery. Descriptive statistics of perception and practice variables and cross tabulation of selected variables was performed.

**Results:**

Most mothers knew that HIV could be transmitted through breast milk. The majority perceived any type of breastfeeding as beneficial to the infant: 230 (83.6%) in private facilities, and 188 (68.4%) public facilities. Over three-quarters of the mothers breastfed their infants and their reasons for breastfeeding included personal choice, cultural norms, fear of HIV status being disclosed and pressure from family members. A statistical significant association was found between; (i) practice of breastfeeding and marital status, (*p* < 0.01), and (ii) practice of breastfeeding and household income provider (*p* = 0.02). However, neither marital status (AOR 1.4; 95% CI 0.3, 6.8) nor being the household income provider (AOR 4.9; 95% CI 0.6, 12.9) is a significant predictor of breastfeeding of HIV-exposed infants.

**Conclusions:**

Breastfeeding remains a common trend among HIV positive women and it is associated with economic independence of women and social support. Fear of stigma negatively affects practice of breastfeeding. Hence, HIV positive mothers need economic independence and the support of family members to practice recommended infant feeding options.

## Background

Human breast milk is the ideal food for human infants [1]. It offers nutrients that are essential for normal growth and proper development, and is central to establishing the foundation for a long healthy life [2, 3]. The antibodies contained in breast milk, which mothers transfer to their infants, act as first line defense against some childhood killer diseases [4]. This mother-to-child transfer of antibodies is considered as passive immunisation. Breastfeeding is also of immense benefit to the mother. When exclusively done, it could be a natural method of delaying pregnancy. This is called lactational amenorrhea method (LAM); a form of contraception that has wide cultural acceptability [5]. Mother to Child Transmission (MTCT) of HIV, also known as vertical transmission, remains the major route of pediatric HIV infection in sub-Saharan Africa [5–7]. Mothers who are HIV positive can transfer the virus to their infants during pregnancy, labour, delivery and breastfeeding [8]. Although it is reported that progress towards the elimination of MTCT is one of the greatest successes of global AIDS response, with MTCT rates of under 5% in some priority countries, there are still challenges with breastfeeding transmission in countries where coverage of antiretroviral treatment (ART) among pregnant women is very low [9].

Infant feeding by HIV-infected mothers is a controversial issue that constitutes global public health dilemma [8]. It is particularly challenging because available options, whether to breastfeed or not, both involve risks to the child that affect their health and survival. Weighing these risks against benefits generates intense debates among policymakers, program managers and service providers in sub-Saharan Africa. This is particularly so when considering that the major causes of infant death, malnutrition and infectious diseases, could be prevented if mothers breastfeed their babies. Whereas breastfeeding involves a small risk of HIV transmission, not breastfeeding poses a considerable risk to infant survival [10, 11].

The recommendation for breastfeeding among HIV mothers has changed over time. Initially, it was recommended that HIV positive mothers should practice exclusive breastfeeding with early and rapid cessation (at 6 months) and exclusive replacement feeding with infant formula or modified cow’s milk [12]. This was replaced by the WHO 2010 recommendation [13], and later by the 2016 guidance for infant feeding and HIV which recommends that mothers known to be HIV should exclusively breastfeed their infants for the first 6 months of life, and can continue breastfeeding with complementary feeds up till 24 months of life [14]. The latter are more in line with public health recommendations for all mothers, as well as customary infant feeding practices in sub-Saharan Africa. This also protects HIV positive women from suspicion and stigma attached to non-normative infant feeding practices [15]. Although the decision to breastfeed an infant is not solely made by the mother, she has the upper hand in determining whether or not to follow through with any decisions and her actions are influenced by her perceptions.

The purpose of this study was to examine the perception and practice of breastfeeding among HIV positive mothers receiving care in public and private facilities that offer comprehensive HIV care and PMTCT of HIV.

## Methods

### Study setting

The study was conducted in 2015 in the metropolis of Enugu city, capital of Enugu state. Enugu is located in the Southeast geopolitical zone of Nigeria [16]. Women and children constitute about 62% of the population [16]. The prevalence of HIV among pregnant women attending antenatal clinic in Nigeria was estimated as 3% [17]. According to the UNAIDS 2017 report, 32% of HIV positive pregnant women had access to ART in 2016, and there were 37,000 new infections among children [9]. It has been reported that MTCT rate is about 32% in Nigeria [18].

A range of public, private and faith-based health facilities serve as key sources of maternal and child health care delivery (Uzochukwu BSC, Onwujekwe OE, Soludo E, Nkoli E, Uguru NP, The District Health System in Enugu State, Nigeria: An analysis of policy development and implementation, unpublished). PMTCT services are offered in some of these facilities in the State. However, only two public and two private facilities were offering comprehensive PMTCT services at the time of the study.

All four facilities were included in this study and they are, for public facilities, University of Nigeria Teaching Hospital (UNTH) and Enugu state University Teaching Hospital (ESUTH); and for private facilities, Annunciation specialist Hospital and Mother of Christ specialist Hospital. PMTCT services are offered daily by skilled health workers (doctors and nurses).

### Care of pregnant and postpartum women living with HIV in Nigeria

Comprehensive PMTCT services consisting of HIV testing for mothers, antiretroviral treatment for mothers, post-exposure prophylaxis for infants, Polymerase chain reaction (PCR) testing for infants and infant feeding counselling are offered to pregnant women and mothers living with HIV in Nigeria. These services are offered in line with the Nigerian National PMTCT guideline [19] and the 2010 WHO guideline for HIV and infant feeding, which also provide for women living with HIV to choose to breastfeed with maternal or infant antiretroviral cover [13].

### Study aim and design

The aim of the study was to examine the perception and practice of breastfeeding of HIV-exposed infants among HIV positive mothers receiving comprehensive care for PMTCT in public and private hospitals in Enugu metropolis.

The study used a cross-sectional descriptive design and HIV positive mothers were interviewed as they exited the final point of service delivery for the day.

### Study population

The study population consisted of HIV positive women receiving care for PMTCT during pregnancy, childbirth and postnatal care. Additionally, women who had babies in the 12 months preceding the study and were still receiving care for PMTCT were included in the study. This is because PMTCT services are provided to mothers until 12 months after delivery, when they are either transferred to adult ART clinic, if they do not become pregnant in the period, or remain in the PMTCT clinic, if they become pregnant.

### Sample size and sampling technique

In order to achieve a power of at least 80% with 95% confidence using a prevalence value of 11% as utilization of PMTCT services, a minimum sample size of 500 was calculated and increased to 550 to account for possible incomplete or non-response of 10%. Proportionate method was used to allocate women to be sampled from each facility and participants were selected consecutively as they exited the PMTCT clinic. Table 1 shows the number of women on PMTCT in each facility and the samples selected. The formula used to calculate sample size per facility is as follows:

**Table 1:** Proportionate allocation of sample selected per facility

Health facility	Number of mothers receiving care for PMTCT	Sample selected for the survey
UNTH	268	154
ESUTH	210	121
Annunciation	224	145
Mother of Christ	202	130
TOTAL	904	550

$$ Sample\ allocation\kern0.75em to\ facility\ A=\frac{No. of\ mothers\ receiving\ PMTCT\ care\ in\ facility\ A}{No.\kern0.5em of\ mothers\ receiving\ PMTCT\ care\ in\ the\ 4 facilities\kern0.5em \ (904)}\ast Sample\ size\ (550) $$

### Data collection method

Pre-tested interviewer administered questionnaires were used to collect information on demographic characteristics of respondents, perception and practice of any type of breastfeeding disaggregated by type of facility (public vs private). Information was also obtained on their individual perception of positive HIV status, as well as their family and community members’ perceptions and support for HIV positive people. The questionnaire was translated to the local language, and validated through pre-testing and expert review.

Data was collected between February and July 2015 by trained field workers and health workers (nurses) working in the PMTCT centers.

### Data analysis

Descriptive statistics was performed using Statistical Package for Social Sciences (SPSS) software version 20. Frequencies and proportions were calculated for categorical variables while means were calculated for numeric variables. Demographic characteristics and perception of HIV status were cross-tabulated with clients’ practice of infant breastfeeding. Chi square test was used to examine the association between, (1) practice of infant breastfeeding and respondents’ demographic characteristics, and (2) practice of infant breastfeeding and perception of HIV positive status. Statistical significance was reported if *p* value was less than 0.05. Logistic regression analysis was done to identify the determinants of breastfeeding among HIV positive mothers. The independent variables that had statistically significant association with practice of breastfeeding at *p* value of less than 0.05 were included in the model.

## Results

A total of 550 HIV positive mothers participated in the study, 275 from public facilities and 275 from private facilities. All 550 questionnaires were analysed and the results are presented in Tables 2, 3, 4, 5, 6 and 7.

**Table 2:** Demographic characteristics of respondents

Variables	Total *n* (%) *n* = 550	Private *n* (%) *n* = 275	Public *n* (%) *n* = 275	χ^2^(*p* value)
Age category (years)				
≤ 24	20 (3.6)	12 (4.4)	8(2.8)	14.39 (0.33)
25–29	177 (32.2)	88 (32.0)	89(32.4)	
30–34	268 (48.7)	144 (52.4)	124 (45.1)	
≥ 35	85 (15.5)	31 (11.3)	54 (19.3)	
Any formal education	525 (95.4)	260 (94.5)	265 (96.4)	0.60 (0.44)
Highest level of education				
Primary	91 (16.5)	41 (14.9)	50 (18.2)	15.14 (0.06)
Secondary	265 (48.2)	121 (44.0)	144 (52.4)	
Tertiary	169 (30.7)	98 (35.6)	71 (25.8)	
Employment status				
Unemployed	176 (32.0)	109 (39.6)	67 (24.4)	3.44 (0.49)
Paid employment	101 (18.4)	55 (20.0)	46 (16.7)	
Self-employed	273 (49.6)	111 (40.4)	162 (58.9)	
Marital status				
Unmarried	74 (13.5)	40 (14.5)	34 (12.4)	0.11 (1.0)
Currently married	476 (86.5)	235 (85.5)	241 (87.6)	
Parity				
1–3	367 (66.7)	203 (73.8)	164 (59.6)	0.01 (0.91)
4–6	183 (33.3)	72 (26.2)	111 (40.4)	
Household income provider				
Partner only	344 (62.5)	170 (61.8)	174 (63.3)	5.01 (0.87)
Self only	117 (21.3)	56 (20.4)	61 (22.2)	
Partner and self only	82 (14.9)	46 (16.7)	36 (13.1)	
Other Relatives	7 (1.3)	3 (1.1)	4 (1.4)

**Table 3:** Perception of HIV positive status

Variables	Total *n* (%) *n* = 550	Private *n* (%) *n* = 275	Public *n* (%) *n* = 275	χ^2^(*p* value)
Self-perception of HIV status				
Feels^a^ inferior to HIV negative peers	290 (52.7)	131 (47.6)	159(57.8)	0.31 (0.58)
Feels equal to peers who are HIV negative	260 (47.3)	144 (52.4)	116 (42.2)	
Family members’ perception of status				
Family member treats self differently because of HIV status	445 (80.9)	209 (76.0)	236 (85.8)	0.07 (0.79)
Family member associates with self regardless of HIV positive status	105 (19.1)	66 (24.0)	39 (14.2)	
Community perception of HIV status				
Community excludes/isolates HIV positive people	478 (86.9)	236 (85.8)	242(88.0)	0.39 (0.53)
Community accepts HIV positive people	72 (13.1)	39 (14.2)	33(12.0)	

^a^ Inferior in terms of self-worth or value

**Table 4:** Perception and Practice of any type of breastfeeding disaggregated by type of facility

Variables	Total *n* (%) *n* = 550	Private *n* (%) *n* = 275	Public *n* (%) *n* = 275	χ^2^(*p* value)
Knows HIV can be transmitted through breast milk	356 (64.7)	184 (66.9)	172 (62.7)	1.69 (0.19)
Perceives breastfeeding of HIV-exposed infant as beneficial	418 (76)	230 (83.6)	188 (68.4)	2.79 (0.09)
Breastfed last child	428 (77.8)	221 (80.4)	207(75.3)	3.95 (0.05)
Reasons for breastfeeding				
Cultural norm	213 (38.7)	106 (38.5)	107 (38.9)	0.10 (0.75)
Personal choice	255 (46.4)	149 (54.2)	106 (38. 5)	0.37 (0.54)
Fear of status being disclosed	97 (17.6)	30 (10.9)	67 (24.4)	0.02 (0.89)
Husband insisted	48 (8.7)	29 (10.5)	19 (6.9)	0 00 (1.00)
Other family member insisted	23 (4.2)	16 (5.8)	7 (2.5)	0.02 (0.88)
Length of time of breastfeeding				
< 6 months	200 (36.4)	79 (28.7)	121 (44. 4)	0.36 (0.55)
≥ 6 months	350 (63.6)	196 (71.3)	154 (56.6)

**Table 5:** Relationship between respondents’ demographic characteristics and practice of breastfeeding

Demographic variables	Breastfed last child			
	Private, *n* (%)	χ^2^(*p* value)	Public, *n* (%)	χ^2^(*p* value)
Age category (years)				
≤ 24	12 (100)	6.62 (0.14)	6 (75.0)	10.4 (0.67)
25–29	64 (72.7)		63 (70.8)	
30–34	119 (82.6)		97 (78.2)	
≥ 35	24 (82.8)		41 (75.9)	
Level of education				
No formal education	12 (80.0)	0.01 (1.00)	10 (100)	10.5 (0.86)
Primary	33 (80.5)		36 (72.0 0	
Secondary	97 (80.2)		113 (78.5)	
Tertiary	79 (80.6)		48(67.6)	
Employment status				
Unemployed	88 (80.7)	1.64 (0.44)	53 (76.)	2.2 (0.71)
Paid employment	41 (74.5)		33 (71.7)	
Self-employed	92 (82.9)		121 (75.6)	
Marital status				
Unmarried	32 (55.6)	12.69 (< 0.01)*	23 (69.7)	0.6 (0.44)
Currently married	189 (80.4)		183 (75.9)	
Parity category				
1–3	171 (84.2)	7.37 (0.21)	116 (71.6)	3.3 (0.15)
4–6	50 (69.4)		91 (80.5)	
Household income provider (s)				
Partner only	139 (81.8)	4.55 (0.41)	136 (78.2)	14.1 (0.02)*
Self only	46 (82.1)		36 (59.0)	
Partner and self only	36 (73.5)		35 (87.5)	

*%* percent of the group

*Refers to *p* values of less than 0.05 which imply statistical significance

**Table 6:** Relationship between practice of breastfeeding and perceptions of HIV positive status

Perception of HIV status	Breastfed last child			
	Private *n* (%)	χ2(*p* value)	Public *n* (%)	χ2(*p* value)
Self-perception of HIV status				
Feels inferior to HIV negative peers	100 (76.3)	2.57 (0.11)	115 (55.6)	1.76 (0.19)
Feels equal to peers who are HIV negative	121 (84.0)		92 (79.3)	
Family members’ perception of status				
Family member treats self differently because of HIV status	164 (78.5)	1.98 (0.16)	174 (84.1)	2.01(0.40)
Family member associates with self regardless of HIV positive status	57 (86.4)		60 (90.9)	
Community perception of HIV status				
Community excludes/isolates HIV positive people	188 (79.7)	0.52 (0.47)	179 (73.9)	5.2 (0.70)
Community accepts HIV positive people	33 (84.6)		18 (85.7)

**Table 7:** Predictors of practice of breastfeeding

Variables	AOR	95% CI of AOR	*p*-value
Public			
Household income provider (s)			
Partner only	2.1	0.7, 5.6	0.13
Self only	4.9	0.6, 12.9	0.08
Partner and self only	1		
Private			
Marital status			
Currently married	1.4	0.3, 6.8	0.70
Unmarried	1		

Table 2 shows the demographic characteristics of the respondents. The mean age of those receiving care from public health facilities was 30.41 (± 3.25) years, and 124 (45.1%) of them were within the 30–34 years age category. A total of 265 (96.4%) had formal education, of which 144 (52.4%) had secondary level education. More than half of the women, 162 (58.9%), were self-employed, and 174 (63.3%) stated that their partners were the only providers of the household income. A total of 241 (87.6%) were currently married and the highest proportion, 102 (37.1%) had three children.

The mean age of those receiving care from private health facilities was 31.02 (± 3.80) years. Most of them were within 30–34 years age category, 144 (52.4%). A greater proportion had formal education, 260 (94.5%), with 121 (44%) reporting secondary level as their highest level of education. Majority, 111 (40.4%) were self-employed and 170 (61.8%) stated that their partners were the only household income provider. A total of 235 (85.5%) were currently married and the highest proportion, 82 (31.3%), had two children.

Table 3 shows the respondents’ perception of their HIV positive status. In the private health facilities, 131 (47.6%) HIV positive mothers reported feeling inferior to their HIV negative peers and 209 (76.0%) of them felt they were treated differently by family members because of their HIV positive status. The majority of them, 236 (85.8%), also felt excluded/isolated by their communities because of their HIV positive status. In the public facility, 159 (57.8%) HIV positive mothers reported feeling inferior to their HIV negative peers and 116 (42.2%) feel equal to their peers and 236 (85.8%) of them felt they were treated differently by family members because of their HIV positive status. Majority of them, 242 (88.0%), also felt excluded/isolated by their communities because of their HIV positive status.

Table 4 shows respondents’ perceptions and practices of any type of breastfeeding of HIV-exposed infants. Among respondents in the private health facilities, 184 (66.9%) knew that HIV could be transmitted through breast milk. The majority of them, 230 (83.6%) perceived breastfeeding as beneficial to infants. Among 221 (80.4%) who reported breastfeeding their infants, 149 (54.2%) chose to do so for personal reasons, 106 (38.5%) breastfed because it is a cultural norm while 30 (40.9%) women did so for fear of disclosure of HIV status. A total of 45 (16.3%) women were influenced by family members to breastfeed their infants.

Among those receiving care from public health facilities, 172 (62.7%) knew HIV could be transmitted through breast milk and 188 (68.4%) perceived it to be beneficial to their infants. About three-quarters of them, 207 (75.3%) actually breastfed and their reasons for breastfeeding were personal choice 106 (38.5%), cultural norms 106 (38.9%), fear of HIV status being disclosed 67 (24.4%), and pressure from family members 25 (10.4%).

Table 5 shows relationship between respondents’ demographic characteristics and practice of breastfeeding. In the private health facilities, there was an association between practice of breastfeeding of HIV-exposed infant and mothers’ marital status (*p* < 0.01). In the public health facilities, there was an association between practice of breastfeeding of HIV-exposed infant and household income provider (*p* = 0.02).

Table 6 shows relationship between practice of breastfeeding and perceptions of HIV positive status. There was no statistically significant association between the practice of breastfeeding and self, family and community perception of positive HIV status.

Table 7 shows results of analysis of predictors of practice of breastfeeding. For public facility users, being the household income provider did not significantly predict practice of breastfeeding of HIV-exposed infants (AOR 2.1; 95% CI 0.7, 5.5) (AOR 4.9; 95% CI 0.6, 12.9). Similarly, for private facility users, marital status did not significantly predict practice of breastfeeding of HIV-exposed infants (AOR 1.4; 95% CI 0.3, 6.8).

## Discussion

The proportion of HIV positive pregnant mothers who knew that HIV could be transmitted through breast milk was higher than those who did not know. Owing to recent increase in interventions aimed at HIV prevention, more people have become aware of HIV transmission [20]. However, the figures we obtained are still lower than what is expected from mothers who are already receiving care for HIV and PMTCT from health facilities where health workers typically provide basic information on HIV prevention particularly as it relates to PMTCT [21]. Failure to do this results in mothers holding onto their misconceptions or picking up incorrect information that could be harmful to the wellbeing of exposed infants. We also observed that mothers receiving PMTCT care from private health facilities were more aware about HIV transmission through breastfeeding than their counterparts in public facilities. The private health facilities that were selected for this study are known to provide health talks routinely to mothers attending antenatal clinics, including PMTCT. Previous studies that have assessed the level of general health knowledge among mothers receiving maternity care have shown variations based on the type of health care facility [22–24]. Although health education sessions are routinely provided to mothers receiving antenatal care in most public health facilities, overall quality of care in public facilities has been reported to be less than that received in private health facilities [22–24], and this may account for the variation observed in this study.

Practice of breastfeeding among the HIV positive mothers was high as majority of them perceived breast milk as beneficial to their infants, hence they breastfed their infants. Although current PMTCT guidelines promote exclusive breastfeeding as a feeding option for HIV-exposed infants (HEI), at the time of this study, HIV positive mothers were not encouraged to breastfeed in order to prevent transmission of the virus to their infants [25, 26]. Breastfeeding of infants is a social norm among Africans, and some studies have reported that since mothers who failed to breastfeed their babies were suspected of having HIV, many HIV positive mothers choose to breastfeed their infants to prevent this suspicion and the resultant stigma [27–29]. The proportion of respondents among those in public facilities that perceived breastfeeding of HIV-exposed infant beneficial is similar to a study conducted in South Africa that reported 64.9% [25], while lower than 83.6% among those in private facilities. The difference could be due to differences in health education of respondents in the facilities. Our findings concerning practice of breastfeeding among the HIV positive mothers was higher than the study conducted in Ibadan, Nigeria where 93.5% choice of infant feeding was formula [29].

A statistical significant association was found between practice of breastfeeding and some independent demographic variables amongst HIV positive mothers. These variables include marital status and household income provider. Irrespective of the type of facility, the patterns of association between demographic variables and practice of breastfeeding were inconsistent. For instance, the relationship between age and breastfeeding shows all women aged 24 years and younger, and over 80% of those aged 30 years and older breast fed their babies whereas about three-quarters of those aged 25–29 breastfed their babies.

At the time of the study, the policy on feeding practices in Nigeria was an option between exclusive use of infant formula, where sustainability is guaranteed, or exclusive breastfeeding for 6 months and continuing with complementary feeds till 12 months [19]. Currently, there has been a change in the dynamics of infant feeding and breastfeeding is no longer considered a significant risk factor for MTCT of HIV because all HIV positive individuals are now placed on lifelong ARVs [14]. This policy is according to WHO HIV and infant guidelines that was revised in 2016, which recommends exclusive breastfeeding for the first 6 months of life while on ARV drugs and continuing breastfeeding with complementary feeds till 24 months [14]. As such, breastfeeding among HIV positive mothers is now encouraged. With the current policy it is important to examine how these factors may be utilized to increase the practice among these women.

Finally, differences between users of private versus public health facilities are critical to informing intervention for PMTCT. However, the focus of our study was not to compare users of private and public facilities. We therefore recommend that future studies could focus on comparing the differences between users of private and public facilities.

## Conclusions

This paper contributes evidence of factors that influence practice of breastfeeding among HIV positive mothers. This information is valuable for designing interventions aimed at reducing MTCT of HIV in developing countries. This study reveals that breastfeeding remains a common trend among HIV positive women in developing countries and that the practice is strongly associated with fear of stigma in society as well as economic independence of the women. In order to guarantee the wellbeing of HIV positive women in developing countries, they need to be supported by caregivers and community members to make the best choices for their children and themselves.

## Abbreviations

ART: Antiretroviral treatment
ESUTH: Enugu State University Teaching Hospital
HIV: Human Immunodeficiency Virus
LAM: Lactational Amenorrhea Method
MTCT: Maternal to Child Transmission
PMTCT: Prevention of Maternal to Child Transmission
UNTH: University of Nigeria Teaching Hospital
